# Simulation of potential habitat overlap between red deer (*Cervus elaphus*) and roe deer (*Capreolus capreolus*) in northeastern China

**DOI:** 10.7717/peerj.1756

**Published:** 2016-03-21

**Authors:** Wen Wu, Yuehui Li, Yuanman Hu

**Affiliations:** 1 State Key Laboratory of Forest and Soil Ecology, Institute of Applied Ecology, Chinese Academy of Sciences, Shenyang, China; 2 University of Chinese Academy of Sciences, Beijing, China

**Keywords:** Species distribution model (SDM), MaxEnt, Habitat overlap, Anthropogenic disturbance, Deer management, Northeastern China

## Abstract

**Background.** Understanding species distribution, especially areas of overlapping habitat between sympatric species, is essential for informing conservation through natural habitat protection. New protection strategies should simultaneously consider conservation efforts for multiple species that exist within the same landscape, which requires studies that include habitat overlap analysis.

**Methods.** We estimated the potential habitat of cervids, which are typical ungulates in northern China, using the present locations of red deer (*Cervus elaphus*; *N* = 90) and roe deer (*Capreolus capreolus*; *N* = 106) in a Maximum Entropy (MaxEnt) model. Our study area was a human-dominated landscape in the Tieli Forestry Bureau located at the southern slope of the Lesser Xing’an Mountains. We grouped 17 environmental predictor variables into five predictor classes (terrain, habitat accessibility, land cover, vegetation feature, and interference), which were used to build habitat suitability models.

**Results.** Habitat accessibility and human interferences were found to have the strongest influence on habitat suitability among the five variable classes. Among the environmental factors, distance to farmland (26.8%), distance to bush-grass land (14.6%), elevation (13.5%), and distance to water source (12.2%) were most important for red deer, distance to farmland (22.9%), distance to settlement (21.4%), elevation (11.6%), and coverage of shrub-grass (8%) were most important for roe deer. Model accuracy was high for both species (mean area under the curve (AUC) = 0.936 for red deer and 0.924 for roe deer). The overlapping habitat comprised 89.93 km^2^ within the study area, which occupied 94% of potentially suitable habitat for red deer and 27% for roe deer.

**Conclusions.** In terms of habitat suitability, roe deer showed greater selectivity than red deer. The overlapping habitat was mostly located in the eastern mountains. The southwestern plain was not a suitable habitat for deer because it was close to Tieli City. Regarding management measures, we suggest that priority protection should be given to the potential areas of overlapping deer habitats found in this study.

## Introduction

Degradation of natural habitats as a result of landscape fragmentation and habitat destruction was recognized as the main reason for reduction of large mammal populations (Bonnot et al., 2013; Couturier et al., 2014; Jorge et al., 2013; Menard et al., 2014). Many traditional conservation measures target endemic or threatened species, such as the Siberian tiger (*Panthera tigris altaica*) and giant panda (*Ailuropoda melanoleuca*), which coexist in similar habitats with some common species; however, these approaches are insufficient for regional biodiversity conservation. Therefore, we should consider conservation efforts for multiple species that simultaneously exist within the same area (York et al., 2011).

Research that elucidates habitat overlap between or among multiple species can be meaningful and important for such protection strategies. Currently, habitat overlap analysis mainly focuses on the following three kinds of relationships among sympatric species: (1) competitive relationships, in particular between invasive and native species, for example, two snake species native to eastern North America, *Nerodia fasciata* and *N. sipedon*, disturbed declining native amphibian, fish, and reptile populations when introduced to California (Rose & Todd, 2014); (2) symbiotic relationships between species that can exist harmoniously within the same habitat, such as occurs with *Dreissena polymorpha* (zebra mussel) and *D. rostriformis bugensis* (quagga mussel) (Quinn, Gallardo & Aldridge, 2014); and (3) predatory relationships. Despite these efforts, research on habitat overlap is still deficient, especially for target species with similar ecological niches.

The aim of habitat overlap analysis is to reveal the distribution pattern of species (Wilson et al., 2013). Traditionally, it has been difficult to obtain field data for such analyses from mountainous sites or large-scale study areas because of the large amounts of time and effort needed (Stehman & Salzer, 2000). For example, the line transect method is often used in flat or homogenous habitat areas because it is difficult to obtain a sufficient number of samples in mountains or forests. In recent years, with the progress of “3S” technologies—remote sensing, geographical information system (GIS), and global positioning system (GPS)—models have been developed to predict and evaluate the potential distribution areas of target species (Araujo et al., 2011; Brown, 2014; Chitale, Behera & Roy, 2014; Elith & Leathwick, 2009; Fourcade et al., 2013; Vieilledent et al., 2013; Ward & Morgan, 2014). These quantitative models (e.g., species distribution models, SDMs) are valuable tools to assess habitat suitability for species at landscape scales. Among them, the maximum entropy (MaxEnt) model has been used worldwide and shown to have several advantages. First, it has better prediction ability compared with other SDMs (Costa et al., 2010; Elith et al., 2006; Pena et al., 2014). Second, it can achieve high prediction accuracy with sparse species-occurrence datasets and is thus more suitable for studying distributions of endangered wildlife (Silva et al., 2014). Third, it has proven suitable for performing habitat overlap analysis for multi-species (Silva et al., 2014; Quinn, Gallardo & Aldridge, 2014; Holzmann et al., 2015). However, the realized niches of species are always changing because of a variety of environmental factors, such as interspecific interactions and human influences. Therefore, it is difficult to correctly extrapolate SDM results in a realistic environment (Holzmann et al., 2015). Despite this, the MaxEnt model is still the ideal choice for studying overlapping habitat distributions of wildlife when species-occurrence data are lacking.

The Lesser Xing’an Mountains are ideal study areas, comprising more than 30,000 km^2^ of forest area that provides good habitat for wildlife. In this region, cervids are the most common herbivores and they have close relationships with predators and vegetation characteristics. Previous studies reported that the presence of viable populations of deer were essential for the persistence of Siberian tigers and leopards (*Panthera pardus orientalis*) (Tian et al., 2011; Wang et al., in press). Wang et al. (in press) provided the most direct and comprehensive evidence that Amur tigers and leopards were returning to China in large numbers, and the Lesser Xing’an Mountains were potential habitat for the two felids to migrate back to China from Russia, but resettlement was unlikely because of cattle grazing, human disturbances, and deer absence. In recent years, deer populations have faced serious threats due to habitat destruction, which has historically occurred at high intensities (Jiang, Zhang & Ma, 2005).

In the past, four common species of cervids were abundant in the Lesser Xing’an Mountains: moose (*Alces alces*), sika deer (*Cervus nippon*), red deer (*Cervus elaphus*), and roe deer (*Capreolus capreolus*). Based on local forestry workers’ experience, moose (nationally protected animal, Category II) disappeared since the 1990s and sika deer (nationally protected animal, Category I) mainly exist in captivity. Wild cervid populations have now been reduced to red deer and roe deer. The population density of red deer in the Lesser Xing’an Mountains was only 0.1–0.2/km^2^ in 2000. There was a 30–40% reduction in the number of red deer, and a stable roe deer population density from 1990 to 2000 (Liu et al., 2000). Thus, red deer and roe deer were chosen as our target species.

Conservation of these declining populations would be enhanced by understanding potential habitat overlap for cervids. Earlier research in the region showed that the various cervid species have similar behavioral characteristics, dietary habits, and habitat preferences (Jiang, 2004; Li, 2005). However, little information is available on the population distribution and patterns of habitat use for deer. Understanding deer habitat use, especially the overlapping habitat of closely related species, is essential for informing habitat protection approaches. In addition, better understanding the habitat distribution of deer is of particular importance for conservation of Siberian tigers and Amur leopards in northeastern China and the Russian Far East (Tian et al., 2014; Wang et al., in press).

We used a recent species presence location dataset and 17 environmental predictors to simulate the potential distribution of habitat and explore relationships between species presence environmental features using a MaxEnt approach (Phillips, Anderson & Schapire, 2006). The main objectives of this study were to: (1) map the overlapping habitat of red deer and roe deer in a human-dominated landscape; (2) determine which key factors influence the distribution of deer species; and (3) explore the influence of habitat types and potential impacts of human disturbance on habitat suitability.

**Figure 1:**
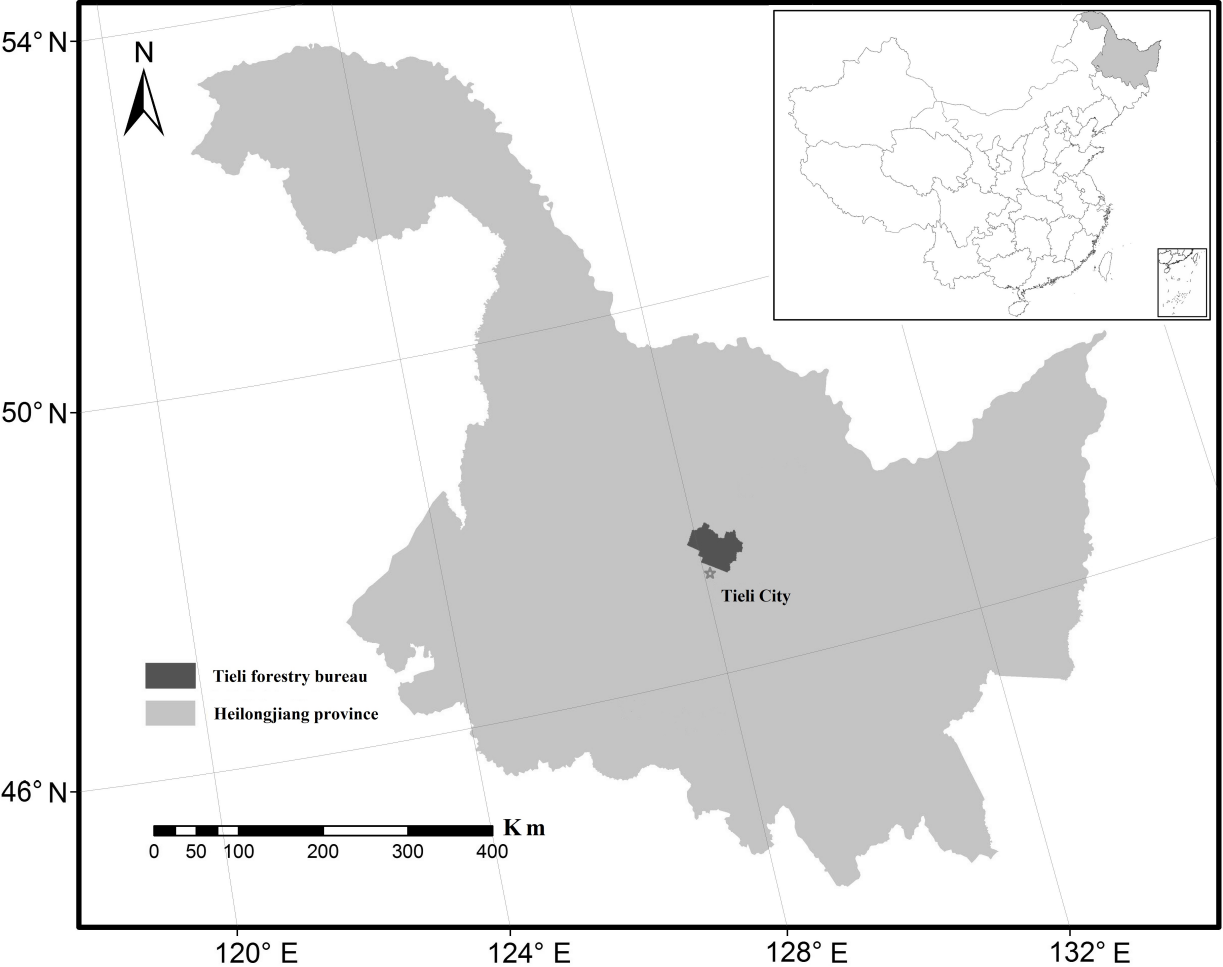
Location of the Tieli Forestry Bureau study area in the Lesser Xing’an Mountains.

## Materials & Methods

### Study area

Our chosen study area was the Tieli Forestry Bureau (TFB) with a total area of 2,058.42 km^2^ (Fig. 1). The TFB is located on the southern slope of the Lesser Xing’an Mountains (47°02′–47°36′N, 127°57′–128°12′E) where forest covers nearly 70% of the landscape. From east to west, the topography changes from mountains to hills, and the elevation ranges from 1,137 m to 224 m a.s.l. The mean summer temperature is 20 °C and the mean winter temperature is −21 °C. Typical broadleaf–conifer mixed forest (Ma et al., 2014) supports more than 100 species of wild animals, such as black bears (*Ursus thibetanus*), red deer, grey cranes (*Grus grus*), mandarin ducks (*Aix galericulata*), and lynx (*Felis lynx*).

In recent years, logging has decreased and was finally banned in 2003. A state-owned forest tenure reform program was initiated in the TFB in 2006. This program involves the selection of commercial forests for management using 50-year contracts with individuals who are designated as land managers at specific sites. The policy has improved the enthusiasm of local foresters and enriched the management patterns of understory planting (Li et al., 2013), but this human pressure disturbed the natural habitats of deer (Li et al., 2014).

### Target species

Characterization of cervid distributions in the TFB is important for three reasons. First, population abundance is very low (0.11/km^2^) because of long-term excessive logging and hunting, and some species have become extinct in several localities (Qin & Zhang, 2009). Second, human activities (e.g., conversions of forest into cropland) have a substantial effect on reducing abundance and distribution of suitable habitat. Third, they play important ecological roles in forest systems, because they are the major food source for predators and represent a large proportion of ungulate species. Therefore, cervids are ideal target species for a case study on SDMs.

### Sampling design and species occurrence data

**Figure 2:**
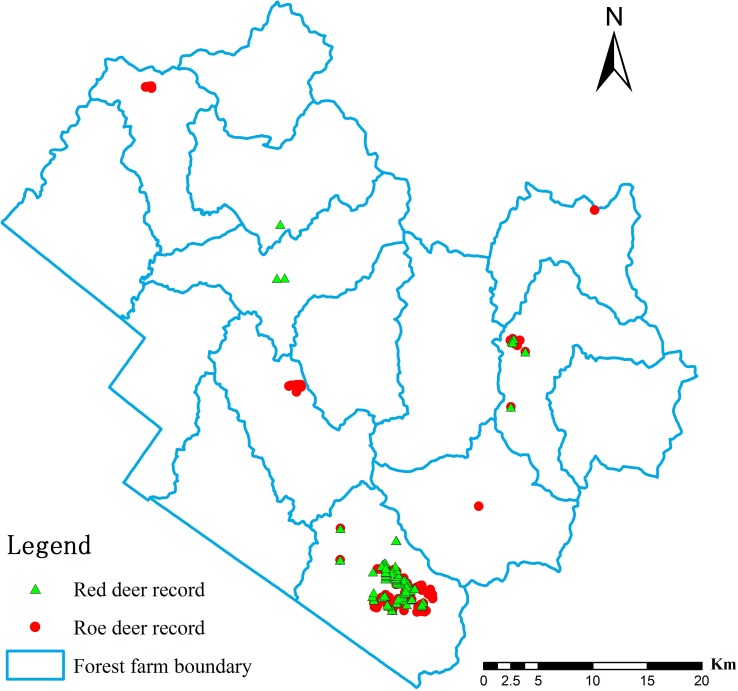
Records of deer presence in the Tieli Forestry Bureau used to model the current distribution of the species.

To collect occurrence records, we used the distance sampling method (Thomas et al., 2010; Waltert et al., 2008) to design “Z” glyph survey routes (1.5–2 km per line, 32 total lines sampled by walking). Along each route, we recorded direct observations, footprints, repose imprints, feces, tracks, and fur, which were clear evidence of deer presence. Depending on observer experience, information about individual deer could be gained based on morphological features such as body size, footprint patterns, and track size. More importantly, a variety of traces can be simultaneously observed in the field, and animal species can be determined based on the specific characteristics of various traces. Field investigations were conducted in the last 3 years (summer 2013, summer and winter 2014, and winter 2015). In total, 196 records were obtained in the field, and their positions were recorded by GPS (90 for red deer and 106 for roe deer, Fig. 2). GPS points were unified in a geographic coordinate system (Beijing_1954) and converted to the format of SDMs as input data within ArcGIS 9.3 (ESRI INC, 2008).

### Species ecology and environmental data acquisition

Red deer and roe deer are two closely related species that occupy nearly the same habitat (e.g., broad-leaved forest and bush-grass land) at a local scale. The suitable habitats of these target species partly overlap and the behavioral ecology and habitat requirements are similar (Jiang, 2007). To estimate the environmental niche, we grouped environmental factors into five major categories: terrain, habitat accessibility, land cover, vegetation feature, and interference factors. No bioclimatic predictors were used because these datasets were usually coarsely scaled (Turner et al., 2003) and more suited to regional or continental SDMs (Elith & Leathwick, 2009; Gillespie et al., 2008).

#### Terrain factors

Terrain variables were derived from a digital elevation model (DEM), which was generated with digital line graphic data (from the National Administration of Surveying, Mapping and Geoinformation of China) at a scale of 1:50,000 and 5-m contour in plains and 10-m contour in mountainous areas. We calculated elevation, slope, aspect, irradiation aspect, surface roughness, and standard deviation of elevation. The irradiation aspect value ranged from 0 to 1 (0 means to accept solar minimum and 1 to accept solar maximum).

#### Habitat accessibility factors

Habitat type data, derived from 3-m-resolution Google Earth imagery (path/row: 117/27, high-resolution Spot 5 satellite images), were used to produce the habitat type map. After investigations in the field, the mean monitoring accuracy was validated as 86%. As a result, habitat type factors included five major habitat types: broad-leaved forest (57%), mixed forest (18%), coniferous forest (15%), farmland (7%), and bush-grass land (2%). By calculating the distance to major habitats, we revealed important habitat accessibility variables, which can reflect the accessibility of different habitats for deer. Additionally, distance to a water source was also taken into account.

#### Land cover factors

We measured land cover classes as the cover of the habitat type map using 1:100,000-scale land use maps. Land cover classes were taken into account because they describe the landscape characterization over a wide geographic range.

#### Vegetation feature factors

Four vegetation feature covariates—forest stand type, forest age, height of tree, and coverage of shrub-grass—were obtained from 1:100,000-scale forest maps, which were sourced from government sectors at the TFB. These four predictor variables clearly reflect the vertical structure and vegetation characteristic of habitats for deer.

#### Interference factors

Human activity data were collected to explore the influence of interference factors on habitat distribution. In our study, interference factors included distance to settlement, distance to road, and distance to forest management area. These human activity-related factors usually had some interference effects, so several buffers were established based on a range of potential impacts. As far as we know, suitable habitat is most strongly affected by visual disturbance, noise, and pollutants, which leads to an animal’s avoidance of roads and settlements. Numerous mammals were reported to suffer higher mortality in close proximity to roads and settlements (Li et al., 2014). Large mammals were shown to move their home range and avoid areas within 100–200 m from roads and 500–1,000 m from settlements (Forman & Alexander, 1998; Trombulak & Frissell, 2000; Li et al., 2014; Zeng, Sui & Wu, 2005), which reduces suitable habitat area. Furthermore, based on our field investigations, we found that the visible distance was about 200 m from the road on both sides. Additionally, human activities generally occur near the residential area within a radius of 500 m. Therefore, we defined buffer zones (200-m width for roads and 500-m radius for settlements) and calculated distances to each of them.

The state-owned forest tenure reform program involves the selection of commercial forests for management at specific sites. Forest workers usually cultivate agaric (*Auricularia auricula*) or ginseng (*Panax ginseng*) at these sites. The policy enriched the management patterns of understory planting. However, with the increase of forestry work intensity and frequency, this human pressure disturbed the natural habitats of deer (Li et al., 2014; Li et al., 2013). Additionally, forest management was taken into consideration because it describes diverse human activities (e.g., understory planting) and produces landscape heterogeneity. Data on forest management areas were drawn from government sectors for the TFB, including size and location information. Because no vector spatial data (e.g., shp. in GIS) were available at such a spatiotemporal scale, our model could not entirely reflect reality. Our solution was to use a circular buffer (equal in area to the size of each specific site) to represent the forest management areas.

Finally, we used ArcGIS 9.3 to interpolate environmental factors into raster-based spatial distribution data and calculated correlation coefficients to avoid the effect of multicollinearity among variables (Dormann et al., 2013). Three pairs of the aforementioned pairwise variables were highly correlated (|*r*| > 0.80) (Hu & Liu, 2014), resulting in a set of 17 environmental predictor variables. Table 1 lists the resolutions, data sources, and calculation methods of these indices.

**Table 1:** Environmental predictors used for modeling. Standard deviation of elevation reflects the roughness of the location.

Factors class	Predictor variables	Initial resolution	Source	Calculation method
Terrain	Elevation	30 m	National Administration of Surveying, Mapping and Geoinformation of China	DEMs
	Slope			
	Aspect			
	Standard deviation of elevation			Statistical analysis
Land cover	Land cover classes	30 m	Land use maps	
Habitat accessibility	Distance to water source	3 m	Google earth imagery	
	Distance to farmland			
	Distance to bush-grass land			
	Distance to coniferous forest			
	Distance to broad-leaved forest			
	Distance to mixed forest			
Vegetation feature	Forest stand type		Forest maps	
	Forest age			
	Coverage of shrub–grass			
Interference	Distance to settlement	3 m	Google earth imagery	500 m buffer zone
	Distance to road			200 m buffer zone
	Distance to management area		Forestry Bureau	Circular buffer zone

### Modeling species distribution

MaxEnt 3.3.3e (http://www.cs.princeton.edu/ schapire/maxent/; Phillips, Anderson & Schapire, 2006) was used to determine a suitable deer habitat. It is currently one of the most commonly used SDM algorithms, and its popularity has increased over the past decade owing to its robust distribution estimates (Elith et al., 2006). We used default values for convergence threshold (10^−5^) and maximum iterations (500), randomly splitting the data into 75% training data and 25% testing data, and 10,000 background points for each MaxEnt run. Area under the curve (AUC) was used to measure model performance and mean area under the receiving operating characteristic (ROC) curve. AUC values ranged from 0.5 (for random probability) to 1 (for perfect ability to predict presence; Phillips & Dudik, 2008). All selected variables were unified a resolution of 90 m, and respective calculations were done in ArcGIS 9.3. Finally, we used these 17 predictor variables to generate two model results: one for red deer and another for roe deer.

### Identifying overlapping habitat areas

To map the overlapping habitat of red deer and roe deer, the Youden index was applied (Vega Garcia et al., 1995) to determine the cut-off point for each model. The ROC curve was obtained by plotting sensitivity versus specificity for various probability thresholds, and the Youden index was calculated by the following formula: sensitivity + specificity—1. Good performance is characterized by a maximal Youden index (Catry et al., 2009; Vega Garcia et al., 1995). Then, we divided the habitat map into suitable (≥threshold value) and unsuitable (<threshold value) habitat based on the maximal Youden index for each species, and assigned 1 for suitable and 0 for unsuitable habitat.

Next, the two reclassified habitat maps were overlapped to generate the final potential habitat suitability map. The pixel value of the map varied from 0 (unsuitable for any species) to 2 (overlapping habitat).

(i) (00) = 0: unsuitable for two species;
(ii) (01 or 10) = 1: suitable for one species but unsuitable for the other;
(iii) (11) = 2: suitable for both species, e.g., the overlapping habitat.

Finally, we calculated the area of major landscape types in the overlapped habitat with the support of ArcGIS 9.3 (ESRI INC, 2008) and R 3.0.1 (http://www.r-project.org/).

**Figure 3:**
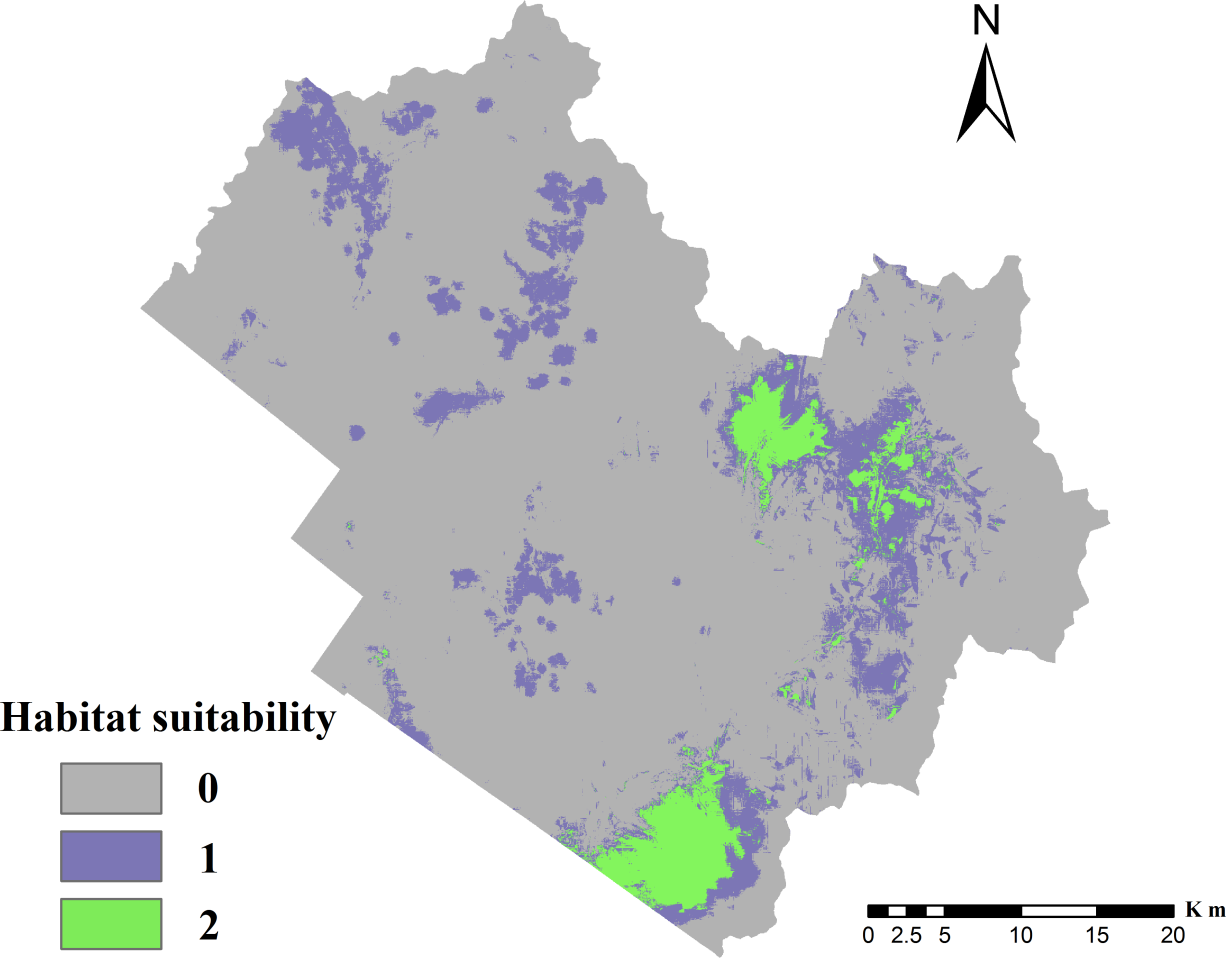
Distribution of suitable cervid habitat. 0, Unsuitable for two species; 1, suitable for one species but unsuitable for the other; 2, suitable for both species, i.e., overlapping habitat.

## Results

### Potential suitable habitat analysis

The testing average AUC for 10 replicate runs of the models was 0.936 ± 0.017 for red deer and 0.924 ± 0.018 for roe deer, indicating that the model predictions were better than chance (AUC = 0.5) and the model could be used to predict the species-occurrence pattern. The optimal cut-off values for red deer and roe deer models were 0.195 and 0.170, respectively. The suitable habitat area was 95.70 km^2^ for red deer and 329.87 km^2^ for roe deer, indicating that roe deer had greater habitat selectivity than red deer in the TFB. The final potential habitat suitability map (Fig. 3) revealed that the unsuitable habitat occupied 1,587.51 km^2^, or 77.7% of the TFB. The overlapping habitat represented 89.93 km^2^ of the study area, which included 94% of potential suitable habitat for red deer. The potential habitat modeled for roe deer constituted 329.87 km^2^, of which 27% also contained overlapping habitat. Results demonstrated that the most highly suitable habitat was located in the eastern mountainous region. The southwestern plain area was not suitable because it was close to Tieli City.

### Environmental factors

**Figure 4:**
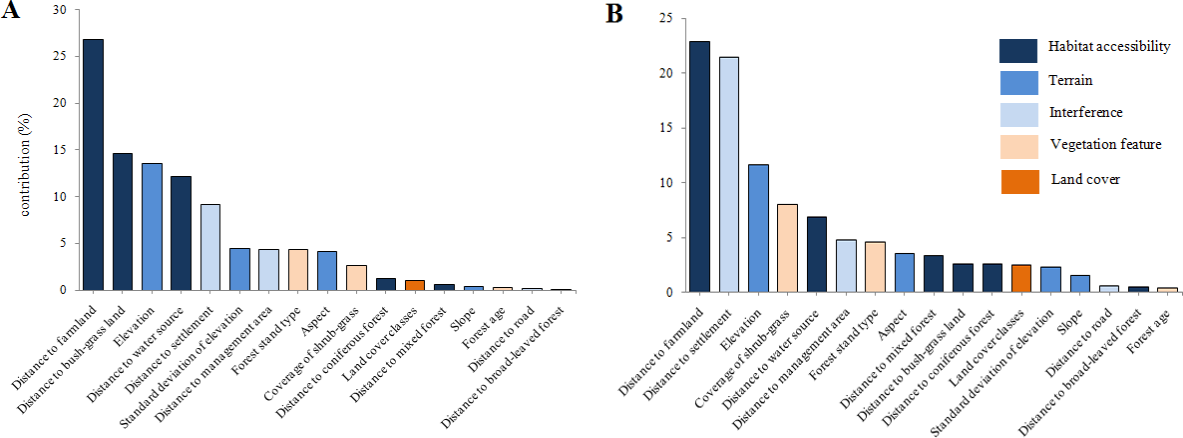
Importance of environmental variables for the MaxEnt models for (A) red deer and (B) roe deer.

Results revealed that the most important environmental variable for predicting potential habitat distribution for red deer was distance to farmland (Fig. 4A; accounted for 26.8% of the distribution) followed by distance to bush-grass land (14.6%), elevation (13.5%), distance to water source (12.2%), and distance to settlement (9.2%). For roe deer, the most important variables were distance to farmland (22.9%), distance to settlement (21.4%), elevation (11.6%), coverage of shrub-grass (8%), and distance to water source (6.9%; Fig. 4B). Out of the above factors, distance to farmland, elevation, and distance to settlement were important variables for both species.

**Figure 5:**
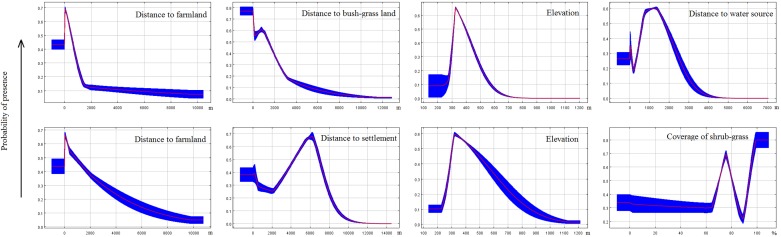
Response curves of MaxEnt models. These curves were generated for the most important variables (the top four in each model, first line for red deer and second line for roe deer) and show the mean response of the cross-validated models with 10 replicate runs (red) and mean ± one standard deviation (blue).

Response curves revealed the direction of the effect of the four most important factors in each model on the potential distribution of deer (Fig. 5). We found that the probability of occurrence decreased in areas with longer distance to farmland. In areas with longer distances to the water source or settlements, the probability of occurrence first increased and then decreased. Occurrence probability sharply increased with increasing distance from a water source (>1.3 km) or settlements (>5.5 km for red deer and 6 km for roe deer). The occurrence probability sharply decreased at a specific elevation (>320 m). We also found that vegetation feature factors contributed little to habitat suitability for deer.

### Proportion of habitat types in suitable habitat classes

**Figure 6:**
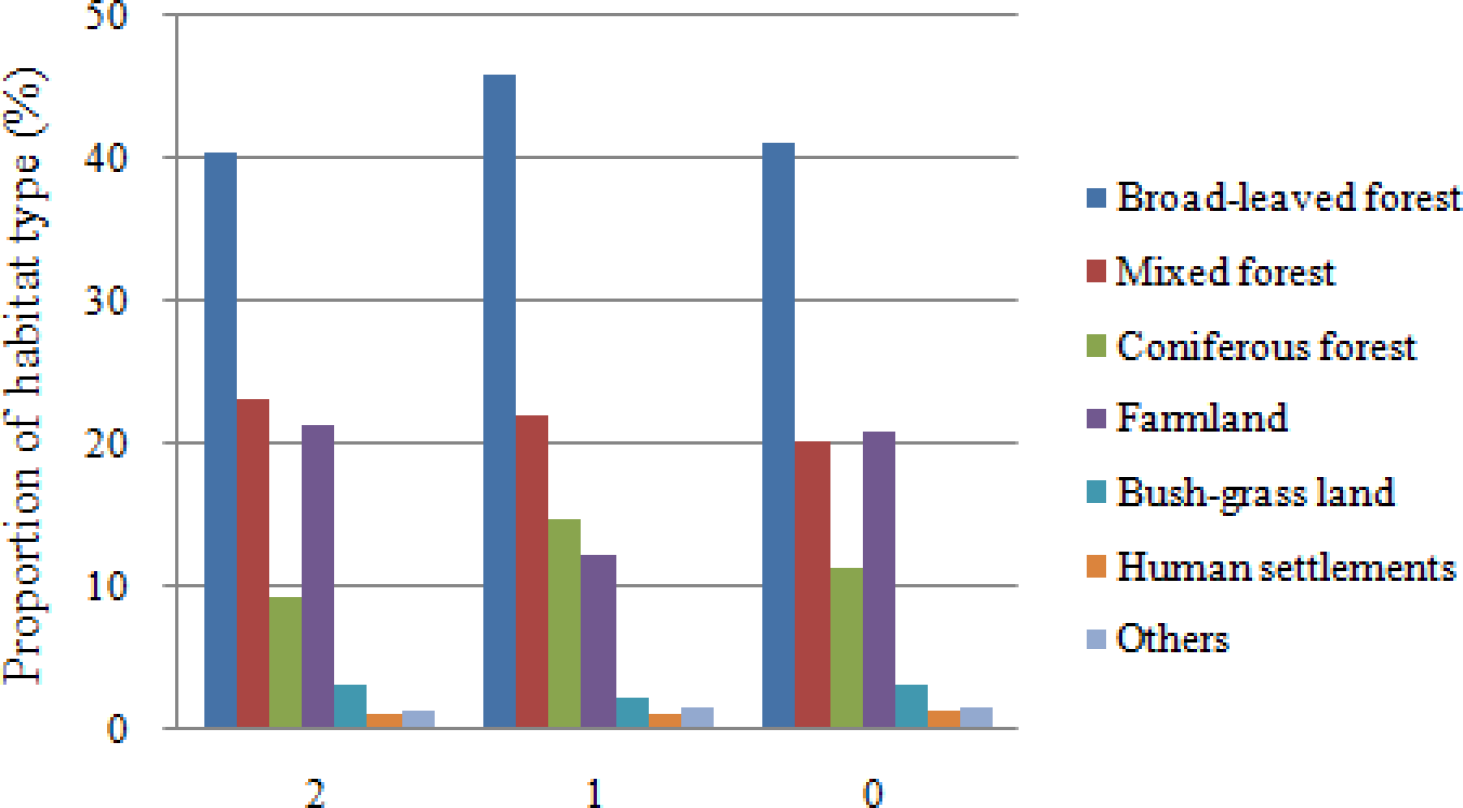
Proportion of habitat types that occur in suitable habitat classes. 0, Unsuitable for two species; 1, suitable for one species but unsuitable for the other; 2, suitable for both species, i.e., overlapping habitat.

Based on raster calculations, comparison of major habitat types in each suitable habitat category showed that farmland was the main habitat type for deer (Fig. 6). It was found that, with a reduced probability of occurrence, farmland covered a fluctuant percentage of areas (21% of overlapping, 12% of suitable, and 21% of unsuitable habitats). This trend was reversed in broad-leaved forests (41% of overlapping, 46% of suitable, and 41% of unsuitable habitats), which is a landscape with low human interference. In general, the overlapping habitat area consisted of a high proportion of broad-leaved forest (41%) and farmland (21%). Broad-leaved forest and mixed forest occupied the main areas of habitat overlap.

## Discussion

### Habitat overlap analysis and environmental factors

The MaxEnt models showed that 27% of roe deer distribution overlaps with predicted red deer distribution, indicating that the niches of the two species are similar. About 94% of the red deer distribution was predicted to be suitable for roe deer, indicating that the realized niche of red deer is smaller. The overlap between the two species in farmland and human settlements was significantly lower than that in broad-leaved forests. This is likely because broad-leaved forests offer deer plentiful food and safety from predators. Indeed, Bonnot et al. (2013) noted that a large portion of deer tend to mostly reside in forests.

The important factors in the models for both species were distance to farmland, elevation, and distance to settlement, indicating that the distributions of both species are limited by similar factors. It is possible that deer distribution is largely affected by distance to farmland, because farmland could provide high nutrition food. We found that the distance to road had little influence on the predicted distribution of deer in this study. Our finding is consistent with those of other studies; for example, Piekielek & Hansen (2012) argued that some roads can be natural wildlife corridors and do not pose a significant disturbance. For terrain factors, Li (2005) also found that altitude and aspect are the main contributors to habitat selection of deer in Heilongjiang Province, China. We found that slope had little impact (with the contributions of 0.4% for red deer and 1.5% for roe deer) on deer distribution, which can be explained by the gentle topography in our study area. In summary, deer prefer the following habitat characteristics: low altitude (about 320 m), far away from residential areas, near farmland and a water source, and suitable coverage of shrub–grass (65–70%). Nevertheless, finer-scale analysis of distribution could present different results; however, this needs to be evaluated with further quantitative research.

### Effects of habitat types on habitat suitability

Broad-leaved forest and farmland are the two most important habitat types selected by deer in our study (Fig. 4), a result that is consistent with those of previous studies. Bonnot et al. (2013) indicated that roe deer prefer living in forests during the day and feeding in farmland at night, which may reduce the risk of being hunted. Behdarvand et al. (2014) found that environmental variables related to anthropogenically influenced land, such as irrigated farms, were most important for predicting wolf-attack risk. We suggest that both habitat types and structures affect deer habitat selection.

### Advantages and limitations

MaxEnt software has proven to be a robust tool in analyses that include current species data with limited biological information (Gogol-Prokurat, 2011; Pena et al., 2014; Phillips, Anderson & Schapire, 2006). In our study, effective occurrence was recorded in field investigations during 2013–2015. Our models performed well when predicting potential distribution, and our findings were more accurate compared with historical data modeling. Past studies supported this view and showed that current species data could improve the accuracy and credibility of results (Brambilla & Saporetti, 2014; Razgour, Hanmer & Jones, 2011; Zeng et al., 2015). Because the accuracy of historical data can be called into question, it cannot be used with confidence in simulations to represent the current distribution state of target species.

However, we admit that this method has limitations, such as that the data sources were not comprehensive enough across the study area. In our study, the sampling was somewhat concentrated near roads, and high elevation areas may have been underrepresented. Such biases may exacerbate statistical problems when models are based on small sample sizes, although our models had robust predictions. In general, it is hard to avoid non-systematic sampling or sampling biases when constructing SDMs (Stolar & Nielsen, 2015). However, Wisz et al. (2008) suggested that MaxEnt had moderate sample size sensitivity combined with excellent predictive ability. MaxEnt was the best and generally outperformed other methods for evaluating low sample sizes. Hu & Liu (2014) pointed out that, although the availability of presence records is a considerable limitation for the application of ecological niche models for threatened species, MaxEnt is recognized to be accurate and stable across all tested sample-size categories.

In addition, we could not fully examine snow as a factor in our study, because its impact was not obvious and data acquisition was difficult on such a spatial scale. However, there have been reports showing that snow becomes an influencing factor for deer at depths greater than 50 cm or in alpine areas (Dou et al., 2013; Ohashi et al., 2014; Ossi et al., 2015). However, in our field investigations, the average snow depth was 5–20 cm and the maximum value was 40 cm. We found that red deer would still feed in suitable foraging areas by pushing the upper snow aside. Generally, we observed that snow did not hinder the daily activities of the deer. Future surveys would benefit from more data, which would allow for better characterization of the suitable overlapping habitat between red deer and roe deer and a better understanding of the factors that shape their distributions. Other important variables to include in future models could be habitat fragmentation and interspecific interactions.

### Management and conservational implications

The Lesser Xing’an Mountain forest has suffered from intense harvesting (including over-hunting of wildlife) since the Japanese invasion in the 1930s. Such a long history of human-driven unsustainable exploitation has resulted in the degradation of wildlife populations and increased sensitivity to disturbance. In addition, many non-forestry land transformations have occurred in the southern TFB areas over the last 30 years, although no quantitative data for landscape change are available at such a spatiotemporal scale. It is obvious that people have been cultivating farmland, building roads and expanding settlements, which have caused the destruction of natural habitats, inevitably transforming the TFB area into a farming–forestry ecosystem. Although recent years have seen few predators and ample food supplies, deer population density has remained very low (Jiang, Zhang & Ma, 2005; Jiang, 2007), a finding we suggest is caused by continued illegal hunting. At the same time, development of agriculture and under-forestry economy (such as understory planting) has led to increasingly isolated large patches, which should be protected. Large herbivores are still very vulnerable to negative anthropogenic effects. For example, results from our roe deer model (Fig. 4B) show that the total contribution of interference factors was 26.8%, which consisted of distance to settlement (21.4%), distance to management area (4.8%), and distance to road (0.6%). In our opinion, the population decline of deer in northeastern China is primarily driven by poaching, which could not be considered in the simulation.

Under conditions of low population density, an understanding of individual species is usually gained by studying habitat distribution. The predicted distribution generated by our study will thus help elucidate the state of cervid habitats. Previous research showed that cervids have similar behavioral characteristics and habitat preferences (Jiang, Zhang & Ma, 2005; Zhang & Zhang, 2010). Jiang (2004) demonstrated that red deer and roe deer feed on the same plant species in winter, whereas red deer and elk (*Elaphurus davidianus*) consistently overlapped with regard to height of vegetation for feeding. These findings indicate that cervids have similar dietary habits. Our research demonstrates that habitat overlap exists between red deer and roe deer. Overlapping habitats of similar species (e.g., red deer and roe deer) might also be used by rare species (e.g., sika deer and elk; Hu & Jiang, 2010).

## Conclusions

Using MaxEnt to study the occurrence of red deer and roe deer allowed us to elucidate habitat suitability within the study area and assess the relative influence of environmental factors on their distribution patterns. Our results demonstrated that the most highly suitable habitat was located in the eastern mountainous region of the TFB, and distance to farmland, elevation, and distance to settlement variables were important for deer. To maintain biodiversity, we suggest that local governments preserve large areas of natural habitat for conservation of multiple species, especially potential overlapping habitats.

## Supplemental Information

10.7717/peerj.1756/supp-1 Supplemental Information 1 Figures data

## Additional Information and Declarations

### Data Availability

The following information was supplied regarding data availability: The raw data is supplied as Supplemental Information.
