# Supplementary figures and images for: Simulation of potential habitat overlap between red deer (Cervus elaphus) and roe deer (Capreolus capreolus) in northeastern China
Source: PeerJ. 2016 Mar 21;4:e1756. doi: 10.7717/peerj.1756 (PMC4806631; doi:10.7717/peerj.1756)

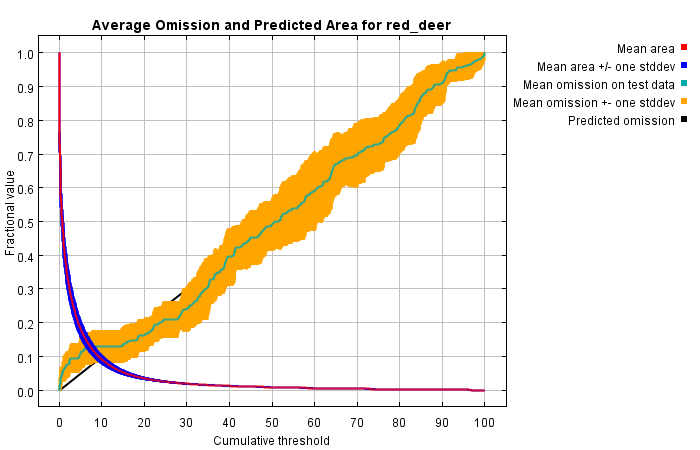

Supplement: Supplemental Information 1 [file peerj-04-1756-s001.zip › Supplemental/red_deer_omission.png]

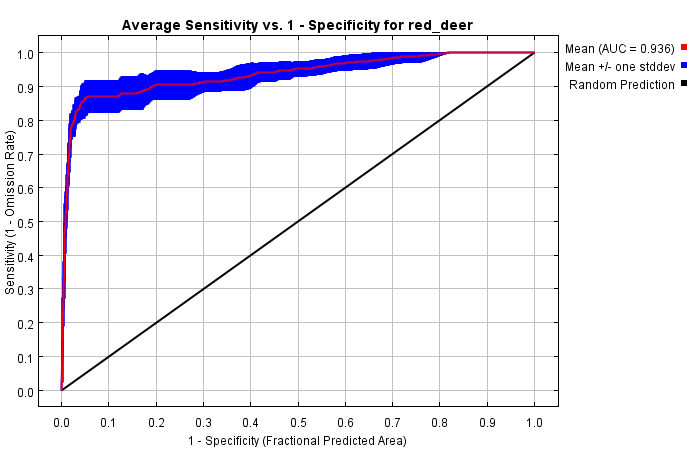

Supplement: Supplemental Information 1 [file peerj-04-1756-s001.zip › Supplemental/red_deer_roc.png]

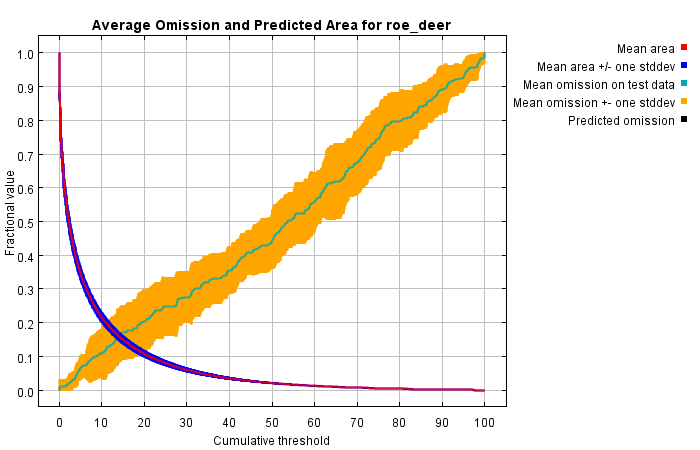

Supplement: Supplemental Information 1 [file peerj-04-1756-s001.zip › Supplemental/roe_deer_omission.png]

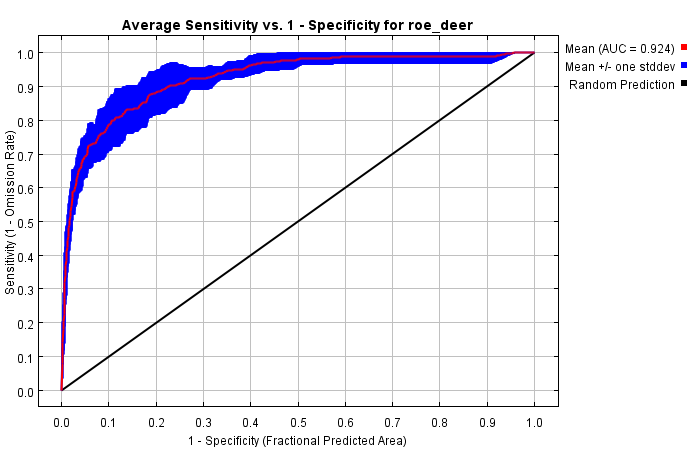

Supplement: Supplemental Information 1 [file peerj-04-1756-s001.zip › Supplemental/roe_deer_roc.png]
